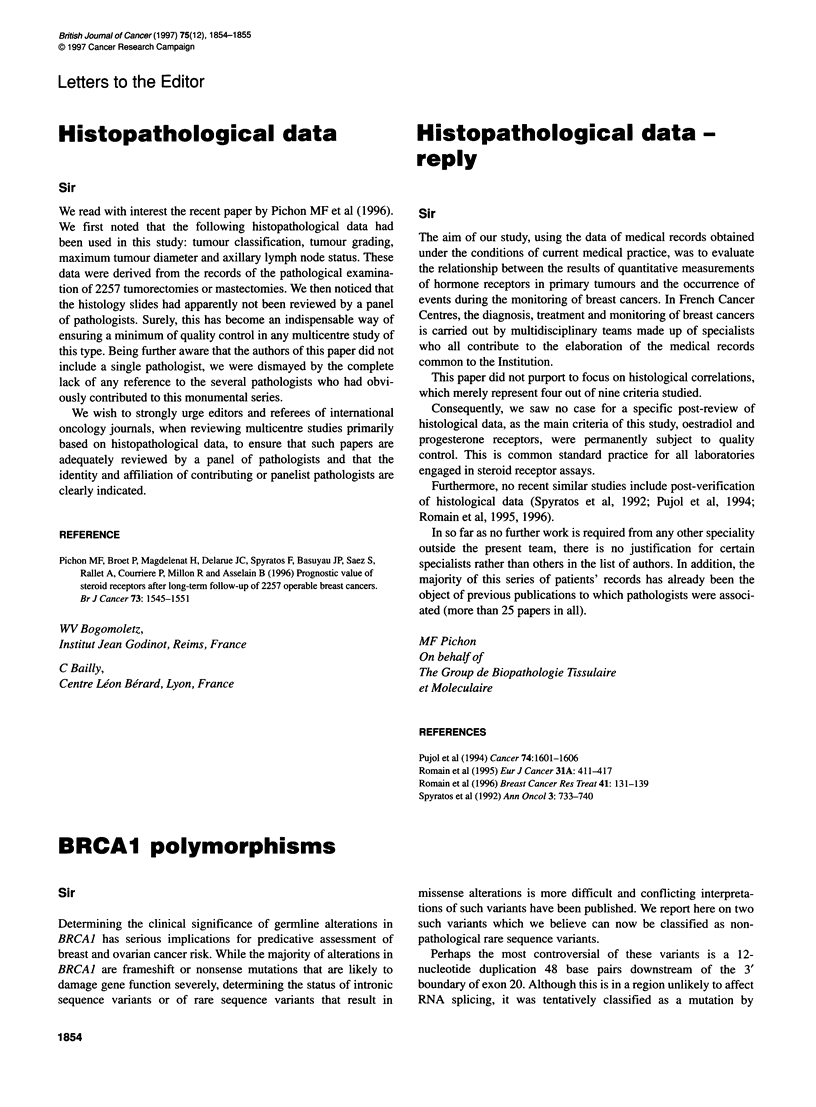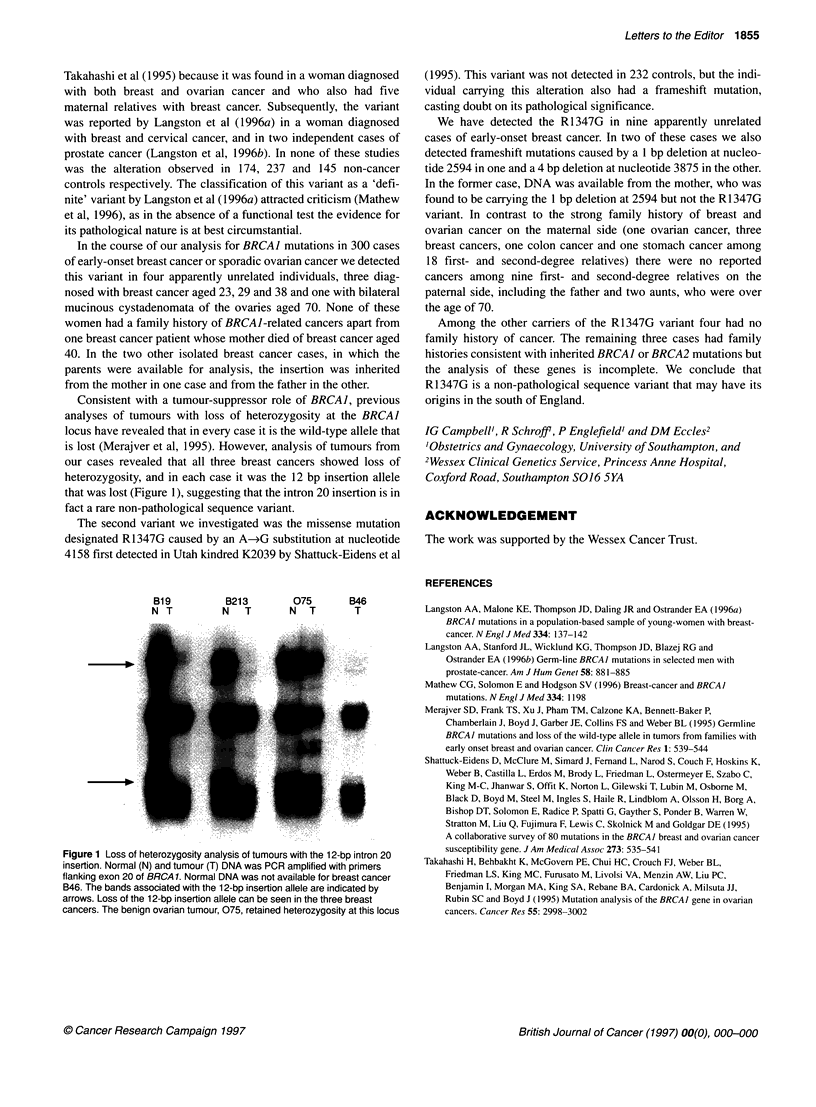# BRCA1 polymorphisms.

**DOI:** 10.1038/bjc.1997.318

**Published:** 1997

**Authors:** I. G. Campbell, R. Schroff, P. Englefield, D. M. Eccles

## Abstract

**Images:**


*British Journal of Cancer* (1997) **75**(12), 1854–1855

© 1997 Cancer Research Campaign

### Letters to the Editor

### BRCA1 polymorphisms

**Sir**

Determining the clinical significance of germline alterations in *BRCA1* has serious implications for predicative assessment of breast and ovarian cancer risk. While the majority of alterations in *BRCA1* are frameshift or nonsense mutations that are likely to damage gene function severely, determining the status of intronic sequence variants or of rare sequence variants that result in missense alterations is more difficult and conflicting interpretations of such variants have been published. We report here on two such variants which we believe can now be classified as non-pathological rare sequence variants.

Perhaps the most controversial of these variants is a 12-nucleotide duplication 48 base pairs downstream of the 3′ boundary of exon 20. Although this is in a region unlikely to affect RNA splicing, it was tentatively classified as a mutation by

Takahashi et al (1995) because it was found in a woman diagnosed with both breast and ovarian cancer and who also had five maternal relatives with breast cancer. Subsequently, the variant was reported by Langston et al (1996*a*) in a woman diagnosed with breast and cervical cancer, and in two independent cases of prostate cancer (Langston et al, 1996*b*). In none of these studies was the alteration observed in 174, 237 and 145 non-cancer controls respectively. The classification of this variant as a 'definite' variant by Langston et al (1996*a*) attracted criticism (Mathew et al, 1996), as in the absence of a functional test the evidence for its pathological nature is at best circumstantial.

In the course of our analysis for *BRCA1* mutations in 300 cases of early-onset breast cancer or sporadic ovarian cancer we detected this variant in four apparently unrelated individuals, three diagnosed with breast cancer aged 23, 29 and 38 and one with bilateral mucinous cystadenomata of the ovaries aged 70. None of these women had a family history of *BRCA1*-related cancers apart from one breast cancer patient whose mother died of breast cancer aged 40. In the two other isolated breast cancer cases, in which the parents were available for analysis, the insertion was inherited from the mother in one case and from the father in the other.

Consistent with a tumour-suppressor role of *BRCA1*, previous analyses of tumours with loss of heterozygosity at the *BRCA1* locus have revealed that in every case it is the wild-type allele that is lost (Merajver et al, 1995). However, analysis of tumours from our cases revealed that all three breast cancers showed loss of heterozygosity, and in each case it was the 12 bp insertion allele that was lost (Figure 1), suggesting that the intron 20 insertion is in fact a rare non-pathological sequence variant.

The second variant we investigated was the missense mutation designated R1347G caused by an A→G substitution at nucleotide 4158 first detected in Utah kindred K2039 by Shattuck-Eidens et al (1995). This variant was not detected in 232 controls, but the individual carrying this alteration also had a frameshift mutation, casting doubt on its pathological significance.

We have detected the R1347G in nine apparently unrelated cases of early-onset breast cancer. In two of these cases we also detected frameshift mutations caused by a 1 bp deletion at nucleotide 2594 in one and a 4 bp deletion at nucleotide 3875 in the other. In the former case, DNA was available from the mother, who was found to be carrying the 1 bp deletion at 2594 but not the R1347G variant. In contrast to the strong family history of breast and ovarian cancer on the maternal side (one ovarian cancer, three breast cancers, one colon cancer and one stomach cancer among 18 first- and second-degree relatives) there were no reported cancers among nine first- and second-degree relatives on the paternal side, including the father and two aunts, who were over the age of 70.

Among the other carriers of the R1347G variant four had no family history of cancer. The remaining three cases had family histories consistent with inherited *BRCA1* or *BRCA2* mutations but the analysis of these genes is incomplete. We conclude that R1347G is a non-pathological sequence variant that may have its origins in the south of England.

**Figure 1** Loss of heterozygosity analysis of tumours with the 12-bp intron 20 insertion. Normal (N) and tumour (T) DNA was PCR amplified with primers flanking exon 20 of *BRCA1*. Normal DNA was not available for breast cancer B46. The bands associated with the 12-bp insertion allele are indicated by arrows. Loss of the 12-bp insertion allele can be seen in the three breast cancers. The benign ovarian tumour, O75, retained heterozygosity at this locus

*IG Campbell[1], R Schroff[1], P Englefield[1] and DM Eccles[2]*

*[1]Obstetrics and Gynaecology, University of Southampton, and*

*[2]Wessex Clinical Genetics Service, Princess Anne Hospital, Coxford Road, Southampton SO16 5YA*

### ACKNOWLEDGEMENT

The work was supported by the Wessex Cancer Trust.

### REFERENCES

Langston AA, Malone KE, Thompson JD, Daling JR and Ostrander EA (1996*a*) *BRCA1* mutations in a population-based sample of young-women with breast-cancer. *N Engl J Med* **334**: 137–142

Langston AA, Stanford JL, Wicklund KG, Thompson JD, Blazej RG and Ostrander EA (1996*b*) Germ-line *BRCA1* mutations in selected men with prostate-cancer. *Am J Hum Genet* **58**: 881–885

Mathew CG, Solomon E and Hodgson SV (1996) Breast-cancer and *BRCA1* mutations. *N Engl J Med* **334**: 1198

Merajver SD, Frank TS, Xu J, Pham TM, Calzone KA, Bennett-Baker P, Chamberlain J, Boyd J, Garber JE, Collins FS and Weber BL (1995) Germline *BRCA1* mutations and loss of the wild-type allele in tumors from families with early onset breast and ovarian cancer. *Clin Cancer Res* **1**: 539–544

Shattuck-Eidens D, McClure M, Simard J, Fernand L, Narod S, Couch F, Hoskins K, Weber B, Castilla L, Erdos M, Brody L, Friedman L, Ostermeyer E, Szabo C, King M-C, Jhanwar S, Offit K, Norton L, Gilewski T, Lubin M, Osborne M, Black D, Boyd M, Steel M, Ingles S, Haile R, Lindblom A, Olsson H, Borg A, Bishop DT, Solomon E, Radice P, Spatti G, Gayther S, Ponder B, Warren W, Stratton M, Liu Q, Fujimura F, Lewis C, Skolnick M and Goldgar DE (1995) A collaborative survey of 80 mutations in the *BRCA1* breast and ovarian cancer susceptibility gene. *J Am Medical Assoc* **273**: 535–541

Takahashi H, Behbakht K, McGovern PE, Chui HC, Crouch FJ, Weber BL, Friedman LS, King MC, Furusato M, Livolsi VA, Menzin AW, Liu PC, Benjamin I, Morgan MA, King SA, Rebane BA, Cardonick A, Milsuta JJ, Rubin SC and Boyd J (1995) Mutation analysis of the *BRCA1* gene in ovarian cancers. *Cancer Res* **55**: 2998–3002

© Cancer Research Campaign 1997